# Time to hospital discharge and subsequent hospital readmission and mortality rates in icu survivors

**DOI:** 10.1186/2197-425X-3-S1-A763

**Published:** 2015-10-01

**Authors:** R Campbell, L Paton, P Stenhouse, A Mackay

**Affiliations:** Victoria Infirmary/ NHS Greater Glasgow & Clyde, Glasgow, United Kingdom

## Introduction

As intensive care mortality falls, there is growing focus on the deficits in physical, mental and cognitive function encountered by survivors of critical illness. Termed the post-intensive care syndrome [1], this significantly and persistently impacts both health-related quality of life [2] and long term survival [3].

## Objectives

We sought to review post-ICU outcomes in patients discharged from our unit to quantify the associated morbidity and mortality that exists following critical illness.

## Methods

Patients surviving to ICU discharge at the Victoria Infirmary, Glasgow, between July 2012 and June 2013 were retrospectively identified and characterised using the WardWatcher^TM^ database.

Subsequent hosptial admissions and mortality over a minimum of one year follow-up were determined using the Clinical Portal^TM^platform and data entered into an Excel^TM^spreadsheet to facilitate analysis.

## Results

During the study period, there were 240 admissions from 231 patients (121 men, 52.4%) where survival to ICU discharge was achieved. This cohort had a mean age of 58 years (IQR 44 - 70) and mean APACHE score of 20 (IQR 14 - 25).

The Majority of ICU admissions were medical (n=143, 61.9%) in comparison to surgical (n=88, 38.1%). Follow-up data indicated 199 patients (86.1%) left hospital at a median of 11 days post-ICU discharge (IQR 5 - 19 days).

Over at least one year, 137 patients (68.8%) were readmitted to hospital on 457 occasions and 24 (12.1%) died.

There was a trend towards reduced survival and longer time to hospital discharge in older patients but no clear effect of age on subsequent hospital admission or mortality rates.

## Conclusions

Our study demonstrates high hospital readmission and significant mortality rates among the proportion of critically ill patients surviving to hospital discharge.

Post intensive care syndrome is a major contributor to these adverse outcomes, but as yet, incompletely understood and inadequately addressed. Intensive rehabilitation may be needed if intensive care is to achieve more than survival, and deliver meaningful patient outcomes with appropriate resource utilisation.

**Table 1:** Follow up data.

	Survival to hospital discharge	Median time to hospital discharge	Subsequent hospital admissions	Subsequent death
Whole cohort (n=231)	199 (86.1%)	11 days IQR [5-19]	137 patients (68.8%) 457 admissions	24 patients (12.1%)
≤ 49 years (n=71)	70 (98.6%)	6 days IQR [2-16]	44 patients (62.9%) 163 admissions	7 patients (10%)
≥ 50 years (n=160)	129 (80.6%)	12 days IQR [7-21]	93 patients (72.1%) 294 admissions	17 patients (13.2%)
≥ 70 years (n=58)	46 (79.3%)	17 days IQR [11.25-27.5]	30 patients (65.2%) 88 admissions	8 patients (13.7%)
≥ 80 years (n=21)	15 (71.4%)	20 days IQR [14.5-27]	10 patients (66.6%) 26 admissions	2 patients (13.3%)
